# P-833. Optimizing Appropriate Transthoracic Echocardiogram Usage in the Setting of Bacteremia

**DOI:** 10.1093/ofid/ofaf695.1041

**Published:** 2026-01-11

**Authors:** Tiffany H Khaw, Paloma Khamly, Iman C Richie, Hannah Chute, Stephanie E Jinno, Susan Bulter-Wu, Ben Lin, Rachel Baden, Devin Clark, Sarah R Freling

**Affiliations:** Los Angeles General Medical Center, Los Angeles, CA; Los Angeles General Medical Center, Los Angeles, CA; USC/LA General Medical Center, Los Angeles, California; Keck School of Medicine, Los Angeles, California; University of Southern California, Los Angeles, California; Keck School of Medicine, University of Southern California, Los Angeles, CA; Los Angeles General Medical Center, Los Angeles, CA; Los Angeles General Medical Center, Los Angeles, CA; Los Angeles General Medical Center, Los Angeles, CA; LA General Medical Center, Los Angeles, California

## Abstract

**Background:**

Transthoracic echocardiogram (TTE) is recommended as part of the initial evaluation for all suspected cases of infective endocarditis (IE). Although there is guidance for ordering transesophageal echocardiograms, when and in whom to obtain TTEs is not clearly delineated. Ordering TTEs when there is low pretest probability for IE can lead to resource over-utilization.

**Figure:**
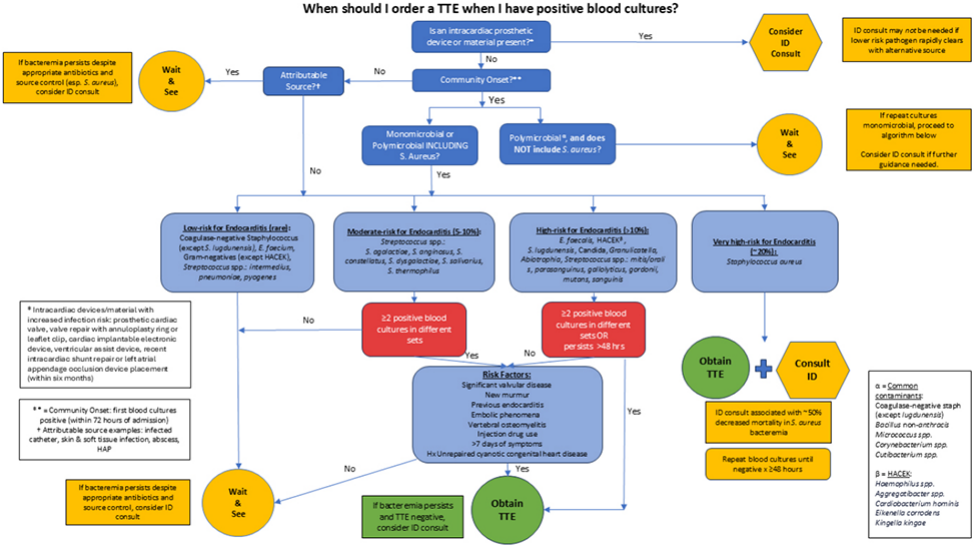
Algorithm for TTE Ordering in Bacteremia Hospital-wide algorithm implemented to assist providers with identifying low, moderate, and high risk features warranting TTE evaluation in the setting of positive blood cultures.

**Figure:**
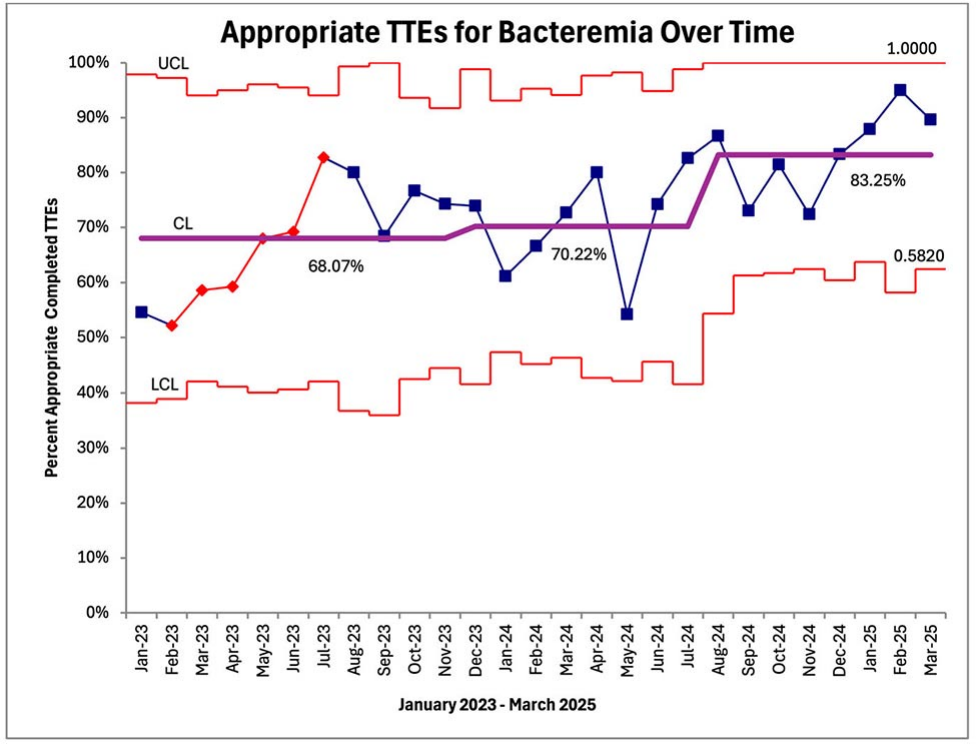
Control Chart of Appropriate TTEs Completed Over Time Control chart demonstrating rise in percent appropriate completed TTEs between January 2023 and March 2025. BCID implemented November 2023. Algorithm implemented August 2024.

**Methods:**

A quasi-experimental study was conducted at Los Angeles General Medical Center that evaluated the appropriateness of TTE orders obtained in patients with bacteremia for the evaluation of IE from January 2023 to March 2025. A hospital-wide algorithm was designed and launched by Infectious Diseases and Cardiology in August 2024 to assist clinicians with the decision to order a TTE in bacteremic patients. The algorithm was created based on previously published data on the likelihood of IE for various organisms and IE predictive scores. Completed orders were manually filtered for those with indications that included “bacteremia”, “positive blood culture”, “vegetations”, or “endocarditis.” Patients included were those with documented bacteremia and age >18 years old. Patients were excluded if they had fungemia or a known diagnosis of IE. TTE orders were deemed appropriate if they met the ordering criteria outlined by the algorithm via chart review.

**Results:**

Baseline data was collected pre-intervention, from January 2023 to July 2024, resulting in 510 patients who met the inclusion criteria. Post-intervention, 203 patients met the inclusion criteria. Notably, the use of rapid blood culture identification (BCID) was reimplemented in our institution in November 2023. Prior to the reimplementation of BCID, the mean appropriateness of TTEs ordered was 68.07%. From November 2023 until July 2024, the mean appropriateness was 70.22%. After the hospital-wide launch of the TTE ordering algorithm and cardiologist review in August 2024, mean appropriateness rose to 83.25%.

**Conclusion:**

Rapid blood culture identification alone only led to a 2% increase in appropriate TTE ordering whereas the implementation of our clinical decision-making tool resulted in a 13% increase. A multimodal approach is imperative to optimize TTE utilization in the evaluation of IE among patients with bacteremia.

**Disclosures:**

Susan Bulter-Wu, PhD , bioMerieux: Honoraria

